# First-Principles Calculations of Oxygen-Dislocation Interaction in Magnesium

**DOI:** 10.3390/ma13010116

**Published:** 2019-12-26

**Authors:** Chao Fang, Jing Zhang, Ying Huang, Jianhao Chen

**Affiliations:** 1 College of Materials Science and Engineering, Chongqing University, Chongqing 400044, China; fangchao01@cqu.edu.cn (C.F.); yinghuang97@outlook.com (Y.H.);; 2 National Engineering Research Center for Magnesium Alloys, Chongqing 400044, China

**Keywords:** magnesium, oxygen, dislocation core, strengthening, first-principles calculation

## Abstract

The interaction between interstitial oxygen atoms and <a>-type screw dislocations was investigated via first-principles calculations to elucidate the effect of oxygen solutes on the deformation behaviors of Mg. The results show that repulsive interactions exist between basal screw dislocation cores and oxygen atoms, which would enable the full basal dislocation to bypass the oxygen atoms in the dislocation glide plane through the cross-slip process. This repulsion also increases the resistance to the motion of dissociated basal dislocations. Moreover, the energy of prismatic <a>-type screw dislocation cores is reduced by the presence of oxygen, which would stabilize the screw dislocation core on the prismatic plane, accordingly facilitating the prismatic slip. This information can complement the fundamental knowledge of alloying Mg using interstitial solutes.

## 1. Introduction

Magnesium has a low density and high strength to weight ratio, which renders it suitable for substituting Fe- and Al-based alloys in the areas of automobile and aerospace [1]. However, the poor formability at room temperature and low strength prohibit the wide application of magnesium [2,3]. Alloying treatment can be an effective method to modify the mechanical properties of Mg. Therefore, a comprehensive understanding of the role of alloying elements on the deformation modes of Mg can assist in the design of novel Mg alloys with improved ductility and higher strength. Numerous efforts have been devoted to illuminating how alloying elements influence the deformation behaviors of Mg [4,5,6,7]. Most available studies concentrate on the substitutional alloying elements. The influence of interstitial solutes, such as nonmetallic elements (C, N, O, and H), on the mechanical properties of magnesium has been seldom regarded. However, the effect of nonmetallic elements in metallic materials should not be neglected. For instance, C atoms can form different phases with Fe atoms, such as ferrite, martensite, and Fe_3_C, leading to various Fe–C alloy systems across a wide range of the strength spectrum. Furthermore, using *ab initio* calculations, Ventelon et al. [8] found that carbon atoms would induce the reconstruction of screw dislocation core in Fe, and therefore be accountable for the solute-segregation related phenomena, such as yielding and strain aging in Fe–C alloy systems. Moreover, in the other two hexagonal close-packed (HCP) metals, Zr and Ti, researchers have discovered that interstitial oxygen atoms would increase the lattice friction against the glide of screw dislocation, thus significantly influencing the plasticity of Zr and Ti [9,10,11]. The aforementioned studies on interstitial nonmetallic alloying elements invoke us to speculate whether the mechanical properties of Mg can be tuned by the solutionization of nonmetallic elements.

In fact, Kang et al. [12] adopted TiO_2_ nanoparticles to alloy oxygen element into Mg metal as interstitial atoms and obtained a Mg-0.3 at.% O alloy with higher yield stress (73 MPa) and a better elongation (5.6%) than that of pure Mg (24 MPa and 3%, respectively). Therefore, improving mechanical properties of Mg through the addition of nonmetallic elements seems to be practically feasible, and it is necessary to study the influence of interstitial alloying elements on the deformation behavior of Mg to complement the fundamental theory of designing novel Mg alloys.

To the best of our knowledge, hitherto only the generalized stacking fault energy (GSFE) of the slip systems (0001) <10-10>, {11-22} <11-23>, and (0001) <11-20> of Mg alloyed with nonmetallic alloying elements have been calculated [13,14], and it is predicted that O and N exhibit an excellent ductilization capability according to Rice criterion [15]. Apparently, these studies are insufficient to clarify the underlying mechanism of the influence of interstitial nonmetallic atoms on the deformation behavior of Mg. In this work, we studied the interaction between O, a representative interstitial nonmetallic solute, and the screw <a> dislocation core in Mg in the scheme of first-principles and analyzed the implication of structural changes of dislocation cores to the mechanical properties of Mg, which would provide additional information on the role of interstitial nonmetallic atoms on the deformation behavior of Mg.

## 2. Computational Method

**Figure 1:**
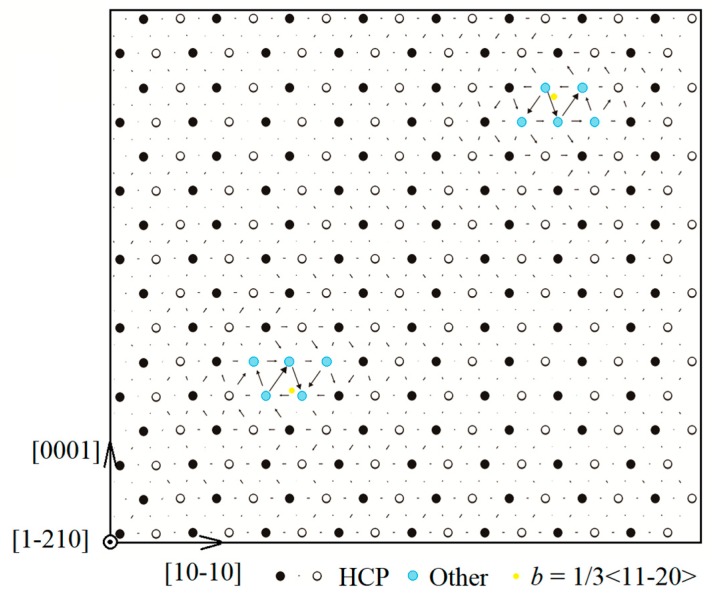
Initial computational supercell with a basal <a> type screw dislocation dipole. The filled and empty circles are colored according to the sequence of <11-20> atomic planes. The magnitude of the arrows in the DD-plots represents the differential displacement of the neighboring rows along [1-210] direction. The center of the dislocation core locates at the closed circuit of arrows or can be represented by the dislocation line (yellow dots), which are perpendicular to the paper. Black/white (filled/empty) and blue sites correspond to hexagonal close-packed (HCP) and other structures, which indicate the perfect lattice and core region in the lattice, respectively. The same convention holds in Figure 2 and Figure 3.

Figure 1 shows the simulation cell consisting of a dislocation dipole with a period quadrupolar arrangement known as an *S* arrangement proposed by Clouet [16], which has been applied to model the dislocation core structure in Zr [10,17] and Mg [18] systems. Three vectors of the simulation box adopted periodic boundary conditions. The periodicity vectors of the supercell are $\vec{U_{1}}$ = 8*a* [10-10], $\vec{U_{2}}=8c\left[0001\right]$, and $\vec{U_{3}}=2a\frac{1}{3}$[1-210] (512 atoms), where *a* (3.204 Å ) and *c* (5.201 Å) are the lattice constants of pure Mg. Two full <a>-type screw dislocations with opposite Burgers vectors on basal planes were constructed by Atomsk code [19] using the isotropic linear elasticity solution. During the above process, a plastic strain was introduced to the simulation box. The unexpected strain was cancelled by the addition of a tilt vector $\vec{\delta }=\vec{b}/2$ to $\vec{U_{2}}$ [16,20]. The dislocation line is parallel to the [1-210] direction. The location and type of dislocations were determined by the dislocation analysis (DXA) in OVITO code [21,22], which could identify the dislocation line and discern HCP, face-centered cubic (FCC), and ‘other structures’ corresponding to the perfect lattice, stacking fault, and dislocation core region in the lattice, respectively. The dislocation lines perpendicular to the paper are denoted by yellow and orange dots for full and partial <a>-type dislocations, respectively. The core structures of the basal screw dislocation can be represented by the atomic displacements close to dislocation cores, which can be visualized in several ways, such as the one proposed by Liang et al. [23] and the differential displacement plots (DD-plots) introduced by Vítek et al. [24]. In the DD-plots, the magnitude of the arrows is determined by the differential displacement of the neighboring rows along [1-210] direction, which can be analyzed with the assistance of Atomsk [19]. The maximum length of the arrows approximately corresponds to *a* and 0.5*a* for full and dissociated <a>-type dislocation, respectively. It is assumed that the dislocation line in DXA pattern and the closed circuit of arrows in DD-plots represent the exact locations of dislocation cores. Two solute atoms were placed at octahedral interstitial sites in the vicinity of each dislocation core of the dipole in the simulation supercell.

All of the geometry optimizations and energy calculations were implemented using the Vienna *Ab initio* Simulation Package (VASP) code [25,26] based on density functional theory (DFT). The generalized gradient approximation (GGA) in the form of Perdew–Burke–Ernzerhof (PBE) [27] was taken to describe the exchange and correlation energy. The energy cutoff of the plane wave energy was set as 1.25 times the largest cutoff energy for each element of interest suggested by VASP (i.e., high precision in INCAR). The first order Methfessle–Paxton with smearing of 0.2 eV was adopted for structural relaxation until the Hellmann–Feynman force on all atoms was less than 10 meV/Å. The extremely accurate energy calculation was then carried out via the linear tetrahedron method with Blöchl correction. The Brillouin zone was sampled using a Monkhorst–Pack *k*-point mesh of 1 × 1 × 16.

## 3. Results and Discussion

**Figure 2:**
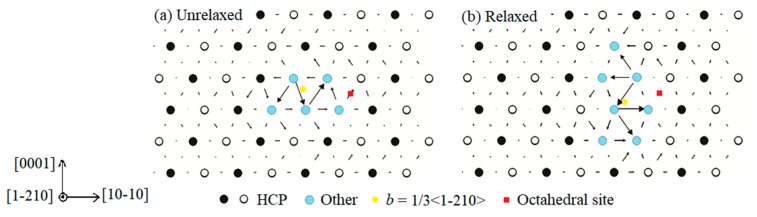
Core structures of full <a>-type dislocation with the introduction of interstitial oxygen atom before and after relaxation. Oxygen atoms were inserted in the octahedral site (Site A) marked as red squares in the vicinity of the dislocation core.

We first studied the influence of O atoms on the core structure of full screw <a> dislocation. The initial unrelaxed core structure in the basal plane is shown in Figure 2a. An oxygen atom was inserted in the octahedral Site A marked as the red square in the vicinity of the basal dislocation core. After ionic and electronic relaxation, screw <a> dislocation almost lay on the prism plane as shown in Figure 2b. This indicates that during the relaxation process, the basal screw <a> dislocation cross-slipped onto the prismatic plane. Normally, in pure Mg, the full <a> dislocation is metastable and would dissociate into two Shockley partials bounded by an ***I***_2_ stacking fault ribbon on the basal plane [18,28]. In this simulation, the dissociation is inhibited under the local influence of an O atom.

**Figure 3:**
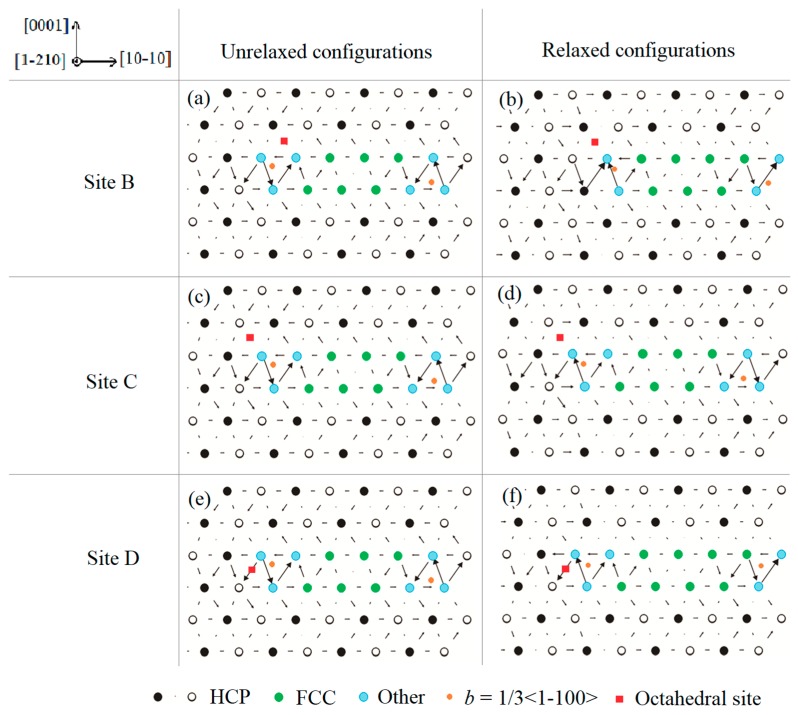
Core structures of dissociated <a>-type dislocation with the introduction of interstitial oxygen atom before and after relaxation. Oxygen atoms were inserted in the octahedral sites marked as red squares in the vicinity of the dislocation core. Green sites correspond to face-centered cubic (FCC) structure of stacking fault (***I***_2_). Orange dots represent the dislocation line of the partial dislocations perpendicular to the paper.

Next, the O atoms were placed in the octahedral sites in the vicinity of the extended basal dislocation core, which was obtained after relaxing the full basal <a> dislocation in pure Mg. The chosen three octahedral sites were labeled as red squares in Figure 3a,c,e. Note that Site B and C are above the glide plane of the dissociated dislocation, and Site D resides in the glide plane. After relaxation, no dramatic structural change is observed. Only when the O atom is located in Site B and D, the extended dislocation cores move a minor distance away from the O atom, which can be seen from the location change of the right partial dislocation line (orange dots) recognized by DXA method in Figure 3b,f. This phenomenon indicates that a repulsive force exists between the O atom and the basal dislocation core. 

To verify this point, the interaction energy between oxygen and screw <a> dislocation core was computed in term of Equation (1) defined as [9,10,29]:

*E*^int^ = (*E*_dislo-O_ − *E*_O_ − *E*_dislo_ + *E*_bulk_)/2 (1)

where *E*_dislo-O_, *E*_O_, *E*_dislo_, and *E*_bulk_ are the energies of supercells with respectively both the dislocation cores and two oxygen atoms, only two oxygen solutes, only two dislocation cores, and no defect nor oxygen solute. The energy of the supercell with dissociated basal dislocation cores before the introduction of oxygen atoms was chosen as the reference energy, *E*_dislo_. A positive value of *E*^int^ indicates a repulsive interaction between a dislocation core and the solute atom.

The values of *E*^int^ for oxygen atoms at Sites A–D are 210, 57, 45, and 63 meV/2*b*, respectively. Positive values imply that these octahedral positions are all repulsive for both full and dissociated dislocation cores in the basal plane. Thus, in actual Mg–O alloys, the oxygen solute atoms would not segregate in the area of the basal screw dislocations. The relaxed core structures of O-decorated dislocations with high energy in Figure 2b only can be formed under applied stress. This phenomenon gives us a scenario that when the basal full <a> dislocation core encounters an interstitial O, basal full dislocation would cross-slip onto prismatic plane to bypass the oxygen atom. The cross-slipping process would induce the creation of jogs that can increase the lattice friction acting against dislocation glide and accordingly strengthen Mg. In Ti– and Zr–O systems this strengthening mechanism has been verified by experimental observations and theoretically simulated [9,10,11,30,31,32]. When a gliding dissociated basal <a> dislocation comes across an interstitial O atom, a larger stress is needed to overcome the repulsive energy between O and the dislocation core, which can strengthen Mg as well. The interactions between oxygen atoms and basal dislocation cores can thus explain the enhanced strength of Mg-0.3 at.% O alloy compared with that of pure Mg [12]. It should be mentioned that this kind of strengthening effect of interstitial O atom in Mg is different from that of substitutional solute atoms. The interactions between most substitutional elements (such as Al, Sn, Y, Gd et al.) and basal dislocation cores are attractive, which indicates those solutes would segregate in the core area and drag the dislocation [18,33].

At last, it is noted that a prismatic dislocation core is formed under the local influence of the oxygen atom as shown in Figure 2b. Compared with the dissociated counterparts in the basal plane, this O-decorated core is of pretty high energy in terms of the value of *E*^int^, which is 210 meV/2*b*. However, if we choose the energy of the supercell with prismatic cores of pure Mg as the reference energy, *E*_dislo_, the interaction energy is −25.9 meV/2*b*. The negative energy indicates an attractive interaction exists between oxygen solutes and prismatic core, and oxygen atoms can reduce the energy of prismatic core. According to Yasi et al. [28], prismatic screw <a>-type dislocation core in pure Mg is of high energy and metastable, which would decompose in the basal plane after relaxation. Thus, the O-decorated prismatic core with lower energy suggests that oxygen can stabilize the screw dislocation core lying on the prismatic plane, which facilitates the prismatic slip. As the important secondary slip system in Mg, prismatic slip can help to alleviate the early fracture of Mg caused by the highly aggregated of basal <a> dislocations during the deformation process, accordingly, advancing the ductility of Mg. This activation of non-basal slip is evidenced by the enhanced plasticity of the Mg–O alloy and the high-resolution transmission electron microscopy (HRTEM) observations of <a>-type dislocation on {1-100} plane in 3%-deformed Mg–O–2Zn sheet [12].

## 4. Conclusions

To elucidate the effect of oxygen solutes on the deformation behaviors of Mg, the interaction between interstitial oxygen atoms and basal <a>-type screw dislocations was calculated utilizing first-principles method. It is found that a repulsive interaction exists between the oxygen atom and basal dislocation cores. This repulsion increases the resistance against the gliding of basal dislocation and contributes to the hardening of Mg containing O solutes. Moreover, the O atom can stabilize the screw <a> dislocation lying on the prismatic plane through decreasing the energy of the prismatic core, which is beneficial to the activity of the prismatic slip. This work provides the basic knowledge of how interstitial oxygen atoms interact with screw dislocation cores and pave the way to design novel Mg alloys.

## Data Availability

The raw and processed data required to reproduce these findings are available to download from http:dx.doi.org/10.17632/k8nfwxsx9p.1.
